# Treating the Synapse in Major Psychiatric Disorders: The Role of Postsynaptic Density Network in Dopamine-Glutamate Interplay and Psychopharmacologic Drugs Molecular Actions

**DOI:** 10.3390/ijms18010135

**Published:** 2017-01-12

**Authors:** Carmine Tomasetti, Felice Iasevoli, Elisabetta Filomena Buonaguro, Domenico De Berardis, Michele Fornaro, Annastasia Lucia Carmela Fiengo, Giovanni Martinotti, Laura Orsolini, Alessandro Valchera, Massimo Di Giannantonio, Andrea de Bartolomeis

**Affiliations:** 1 NHS, Department of Mental Health ASL Teramo, Psychiatric Service of Diagnosis and Treatment, Hospital “Maria SS dello Splendore”, 641021 Giulianova, Italy; 2 Laboratory of Molecular and Translational Psychiatry, Department of Neuroscience, Reproductive and Odontostomatogical Sciences, University of Naples “Federico II”, 80131 Napoli, Italy; felix_ias@hotmail.com (F.I.); lisabuonaguro@tin.it (E.F.B.); adebarto@unina.it (A.d.B.); 3 Polyedra Research Group, 64100 Teramo, Italy; dodebera@alice.it (D.D.B.); dott.fornaro@gmail.com (M.F.); annastasia.fiengo@gmail.com (A.L.C.F.); giovanni.martinotti@gmail.com (G.M.); laura.orsolini@hotmail.it (L.O.); alessandrovalchera@gmail.com (A.V.); 4 NHS, Department of Mental Health ASL Teramo, Psychiatric Service of Diagnosis and Treatment, Hospital “G. Mazzini”, 64100 Teramo, Italy; 5 Department of Neuroscience and Imaging, University “G. d’Annunzio”, 66100 Chieti, Italy; digiannantonio@unich.it; 6 New York State Psychiatric Institute, Columbia University, New York, NY 10027, USA; 7 Casa di Cura Villa San Giuseppe, 63100 Ascoli Piceno, Italy

**Keywords:** Homer, bipolar disorder, schizophrenia, postsynaptic density (PSD), gene expression, transductional pathways

## Abstract

Dopamine-glutamate interplay dysfunctions have been suggested as pathophysiological key determinants of major psychotic disorders, above all schizophrenia and mood disorders. For the most part, synaptic interactions between dopamine and glutamate signaling pathways take part in the postsynaptic density, a specialized ultrastructure localized under the membrane of glutamatergic excitatory synapses. Multiple proteins, with the role of adaptors, regulators, effectors, and scaffolds compose the postsynaptic density network. They form structural and functional crossroads where multiple signals, starting at membrane receptors, are received, elaborated, integrated, and routed to appropriate nuclear targets. Moreover, transductional pathways belonging to different receptors may be functionally interconnected through postsynaptic density molecules. Several studies have demonstrated that psychopharmacologic drugs may differentially affect the expression and function of postsynaptic genes and proteins, depending upon the peculiar receptor profile of each compound. Thus, through postsynaptic network modulation, these drugs may induce dopamine-glutamate synaptic remodeling, which is at the basis of their long-term physiologic effects. In this review, we will discuss the role of postsynaptic proteins in dopamine-glutamate signals integration, as well as the peculiar impact of different psychotropic drugs used in clinical practice on postsynaptic remodeling, thereby trying to point out the possible future molecular targets of “synapse-based” psychiatric therapeutic strategies.

## 1. Introduction

The post-synaptic density (PSD) is a specialized matrix located at excitatory post-synaptic terminals with a disc-shaped aspect, a surface area of 0.07 μm^2^ and a thickness of 30–40 nm at the electron microscopy [1].

The PSD can be described as a macromolecular complex of several hundreds of proteins acting as a molecular switchboard of multiple interacting neurotransmitter signaling pathways [2,3,4].

Results from PSD preparations obtained with different proteomic purification essays have revealed that more than 400 proteins can be regularly found by mass spectrometry fingerprinting in the PSD proteome [5]. These proteins include: membrane receptors and channels, signaling proteins, scaffold and anchoring proteins, GTPases and regulator proteins, kinases, and phosphatases, cytoskeleton proteins [1,6,7].

PSD molecules are involved in several functions critical to dopamine and glutamate-dependent synaptic plasticity processes at glutamatergic synapses [8,9]. Indeed, *N*-Methyl-d-Aspartate (NMDA) receptors represent the core of this protein mesh, whilst non-NMDA ionotropic and metabotropic glutamate receptors are located at the edge of the PSD [1]. Specifically, glutamatergic receptors are targeted at the postsynaptic membrane by PSD multiprotein complexes that regulate their clustering, signal-transduction activity and therefore synaptic rearrangements [10].

Intriguingly, mass spectroscopy evaluations have provided the possibility to compare protein expression with protein phosphorylation data regarding the composition of the PSD proteome obtained from rodent cerebral tissue [11]. It has, therefore, been found that the PSD shows regional differences, especially in terms of protein phosphorylation that is relatively higher in the hippocampus [11]. Moreover, it has been recently reported that microRNAs (miRNAs) precursors can be detected within synaptic fractions tightly associated with the PSD, where they play a role in the direct or indirect regulation of membrane receptors expression [12,13]. Altogether, these findings may reflect regional variations in the molecular mechanisms underlying synaptic plasticity processes in different areas of the brain.

Scaffold molecules at the PSD are major players in the regulation of synaptic plasticity processes. Indeed, by linking the different components of glutamate receptor complexes and by regulating glutamate receptor trafficking, scaffold proteins modulate the signaling cascade starting from membrane receptors and ultimately regulate dendritic structure and function [4,14].

Within the scaffold protein subset, the NMDA receptor and type I metabotropic glutamate receptor (mGluR1/5) scaffold members of the membrane-associated guanylyl kinase (MAGUK) family, the Homer and the ProSAP/Shank (SH3 domain and ankyrin repeat-containing protein) families of proteins are the molecules that have mostly attracted study since the accumulating evidence of their direct involvement in synaptic plasticity processes [4,15].

Proteins containing the PSD-95/disc large/zonula occludens-1 (PDZ) domain are considered a hallmark of the PSD, and the MAGUKs, including PSD-95, SAP102 and PSD-93, comprise three PDZ domains in their N-terminus followed by a src homology-3 (SH3) domain and a guanylate kinase (GK) domain in the C-terminus [16]. PDZ domains are peptide-binding domains, which allow the above-mentioned proteins to interact with a variety of binding partners within the PSD, such as NMDA receptors, as well as cytoplasmic proteins [17]. Moreover, it has been shown that PSD-95 may interact with dopamine and serotonin receptors and regulate their activation state [18,19]. Therefore, MAGUKs participate to the formation of protein complexes within the PSD by assembling in multimers that stabilize membrane receptors and provide a physical link between receptors and intracellular molecules at the crossroad among glutamatergic, dopaminergic, and serotonergic signaling pathways [6,9].

The other well-known family of PSD scaffolds is the ones of the Homers, which is composed by three isoforms in mammals (Homer 1, Homer 2, and Homer 3) and takes part to a variety of biological functions within the PSD [20]. To note, Homer isoforms include two inducible, non-multimerizing splice variants (namely, Homer 1a and Ania-3), which lack the C-terminal domain and are expressed in an immediate-early gene fashion secondary to a variety of neuronal stimuli [20]. Particularly, Homer 1a acts like a dominant negative since it lacks the oligomerization domain and disrupts long Homer-mediated clusters that anchor type I mGluRs to NMDA receptors and bridge mGluRs to their intracellular downstream effectors [21,22]. Therefore, Homers long versus inducible isoforms expression pattern plays a central role in the modulation of the cross-talk between the glutamatergic and different neurotransmitter signaling pathways, in the regulation of intracellular Ca^2+^ dynamics and, ultimately, in dendritic spine remodeling [20,23].

Finally, the ProSAP/Shank family of proteins is composed by Shank 1, Shank 2, and Shank 3, which are considered key organizing PSD scaffolds implicated in modulating glutamate neurotransmission [14,24]. Particularly, Shank proteins allow the formation of polymeric network complexes that require the assembly of Homer tetramers and have been proposed to build functional platforms for other PSD proteins [25].

Indeed, affinity-purified complexes obtained from PSD fractions have been described to include 2-amino-3-(3-hydroxy-5-methylisoxazol-4-yl) propanoic acid (AMPA) receptor subunits (GluR1, GluR2, GluR3, GluR4), subunits of the NMDA receptor (NR1, NR2A, NR2B), G protein regulators and scaffolds such as PSD-95, Shank 2, Shank 3, and Homers [26].

PSD proteins have been crucially involved also in direct interactions amongst membrane receptors. Indeed, growing evidence has been accumulating demonstrating complex connections between glutamatergic and dopaminergic receptors, which implicate imbricated interactions with PSD structures (see [23] for a review). Lee et al. [27], for instance, have reported direct co-immunoprecipitation of NMDA glutamate and D1 dopamine receptors in both hippocampal and striatal cultures. Moreover, the manipulation of either NMDA or D1 receptor may reciprocally influence each other’s functions [28]. However, the scaffolding protein PSD-95 seems to be crucially required for D1 dopamine receptors in order to modulate NMDA currents [29], as well as PSD-95 has been described to directly regulate NMDA functions by abolishing the NMDA-mediated inhibition of D1 receptors internalization [18].

Interestingly, PSD molecules have been implicated also in the fine modulation of transductional pathways starting at dopamine D3 receptors, a subtype of dopamine receptors which has gained increasing interest because of its role in schizophrenia, drug addiction, and antipsychotics mechanisms of action [30]. Peculiarly, the expression of D3 receptors has been demonstrated to be regulated by Brain-Derived Neurotrophic Factor (BDNF) in the nucleus accumbens during development and adulthood [31], a feature that tightly links these receptors to the developmental pathogenetic hypothesis of psychosis and depression, as well as the selective expression of D3 receptors in proliferative zones of striatal granule cells during embryonic development [32].

At the synaptic level, D3 receptors have been found surprisingly located in asymmetric synapses at the head of dendritic spines [33], differently from D1 and D2 receptors that are diffused all over dendrites in striatum, and this peculiar localization suggests direct interaction with NMDA and AMPA glutamate receptors at postsynaptic sites [34]. Indeed, D3 receptors may bind calcium/calmodulin-dependent protein kinase (CaMKII) in the PSD-enriched nucleus accumbens neurons and the activation of NMDA receptors may stimulate D3 function by increasing calcium-dependent CaMKII activation [35]. The modulation of D3 receptors function by PSD is also suggested by a direct impact of D2/D3 selectively binding antipsychotics, such as amisulpride, on the expression of PSD genes [36,37].

Clinical and preclinical studies have extensively provided evidence of abnormal expression and/or functioning of various PSD proteins in diseases such as schizophrenia, bipolar disorder, and autism [9,38,39,40]. These findings are not surprising considering the master role of the above-mentioned molecules in synaptic plasticity processes that are considered aberrant in neuropsychiatric diseases [41].

Due to the studies conducted so far, showing that PSD molecules are modulated by antipsychotics [37,42,43] and play a key role in behavioral conducts [44,45], these molecules are gaining relevant interest as putative targets of pharmacological strategies. However, there are still potential limitations that may apparently prevent large-scale development of PSD protein-targeted therapeutic devices.

Despite strong genetic evidence [46], the specific role of PSD molecules in the pathophysiology (and putatively in therapeutics) of psychiatric diseases is still elusive. One possible hypothesis is that PSD molecules may concur to synaptic pathology in multiple psychiatric conditions, such as autism [47], schizophrenia [48,49], or mood disorders [50]. According to this view, in schizophrenia post-mortem brain tissue an altered expression of proteins belonging to the PSD fraction it has been observed, leading to the conclusion that, within the PSD, NMDA-interacting, and endocytosis-related proteins contribute to schizophrenia pathophysiology [49]. Moreover, transgenic animal models carrying mutations in the genes coding for PSD molecules exhibit clear behavioral phenotypes relevant to psychopathological conditions in psychiatric diseases [51,52,53,54]. Genetic manipulations of PSD molecules cause dendritic spine defects, which are putatively the basis for behavioral aberrations relevant to psychiatric diseases [55,56]. This increasing body of evidence supports the view that PSD proteins are crucially implicated in aberrant synaptic plasticity, and thereby in high-order cognitive alterations, which are the core of pathophysiology in psychiatric diseases.

Here we review the latest studies regarding the role of PSD molecules in the mechanisms of action of the psychopharmacologic drugs mainly used in the treatment of major psychiatric disorders. Moreover, we will explore possible new avenues in the horizon of “PSD-targeting” therapeutic strategies.

## 2. Involvement of PSD Molecules in Psychiatric Drugs Mechanisms of Actions

**Figure 1:**
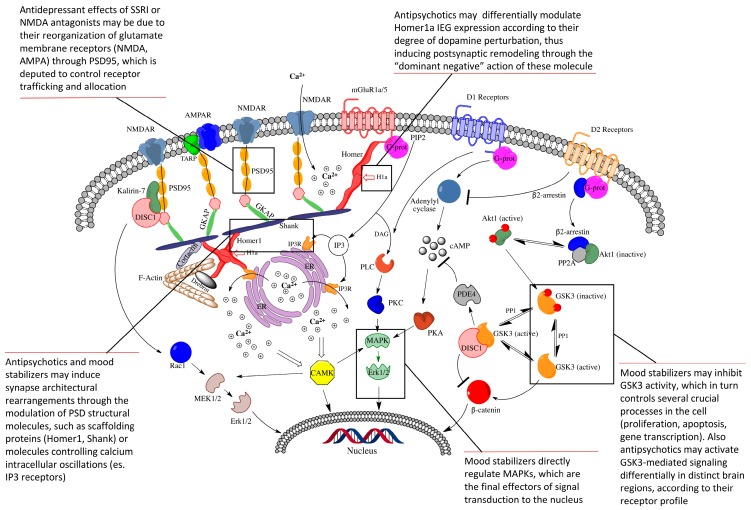
Schematic representation of how postsynaptic density (PSD) proteins elaborate and integrate multiple transductional pathways starting at main dopamine and glutamate membrane receptors. Scaffolding proteins (Homer, Shank, PSD-95) physically connect receptors, linking them to intracellular calcium stores. Transductional pathways activated by dopamine receptors closely interconnect with glutamatergic ones via key PSD proteins, such as GSK3, which elaborates and regulates neuronal survival and differentiation. All transductional pathways route receptors signaling to appropriate nuclear targets via specific effectors, such as CaMK, MAPKs, or Erk, in order to finely modulate long-term activity-dependent neuronal rearrangements. The call-outs describe the impact on some crucial PSD molecules by psychopharmacologic drugs, as discussed in the text. *NMDAR*, *N*-methyl-d-aspartate glutamate receptor; *AMPAR*, α-amino-3-hydroxy-5-methyl-4-isoxazolepropionic acid glutamate receptor; *mGluR1a/5*, metabotropic glutamate receptor type 1a/5; *TARP*, transmembrane AMPA receptors regulating protein or stargazin; *PSD-95*, postsynaptic density protein 95kD; *DISC1*, disrupted in schizophrenia 1; *GSK3*, glycogen synthase kinase 3; *PDE4*, phosphodiesterase 4; *GKAP*, guanylate kinase associated protein; *H1a*, Homer1a immediate-early inducible protein; *PIP2*, phosphatydilinositol bisphosphate; *DAG*, diacylglycerol; *IP3*, inositol 1,4,5-trisphosphate; *cAMP*, cyclic adenosine monophosphate; *ER*, endoplasmic reticulum; *PLC*, phospholipase C; *PKC*, protein kinase C; *PKA*, protein kinase A; *CAMK*, calcium-calmodulin regulated kinase; *MAPKs*, mitogen-activated protein kinases; *Erk*, extracellular signal-regulated kinase; *MEK*, MAPK/Erk kinase; and *Rac1*, Ras-related C3 botulinum toxin substrate 1.

Consistent with the crucial position of postsynaptic proteins at the crossroads of transductional pathways implicated in synaptic plasticity, several studies have demonstrated their central role in the mechanisms of action of drugs currently considered as the mainstay treatment of major psychiatric disorders, such as antipsychotics, antidepressants, and mood stabilizers (Figure 1).

### 2.1. Antipsychotic Drugs

Since the very early studies on the impact of antipsychotic treatment on the brain it was clear that these drugs may induce ultrastructural changes in both cortical and subcortical glutamatergic synapses with significant differences between first generation (FGA) and second generation antipsychotics (SGA) [57,58,59]. Moreover, early studies by de Bartolomeis’s laboratory team demonstrated that both FGAs and SGAs may directly impact the scaffolding proteins that constitute the architecture of PSD via differentially inducing, in both acute and chronic paradigms, the expression of the immediate-early gene *Homer 1a* [60,61]. Particularly, the specific perturbation of dopaminergic signaling may lead to concurrently specific differential topographical brain expressions of Homer family PSD genes directly depending on the receptor profile of the antipsychotic [62], thereby suggesting Homer as a molecular marker of glutamatergic impact by antipsychotics. PSD molecules directly linked to glutamate NMDA receptors, such as PDS95, have been demonstrated to be modified by FGAs and SGAs [63]. More recent studies specifically correlated the impact of SGAs on PSD-95 with their ability to modulate serotonergic neurotransmission together with dopaminergic one [64].

The effects of antipsychotics on PSD molecules are so specific that the topographical pattern of PSD genes expression may vary with the dose of the antipsychotic administered: indeed, increasing doses of selected FGAs or SGAs may progressively recruit the expression of crucial PSD genes, such as *Zif268*, *Homer1a*, *Arc*, and *c-fos*, while gradually impacting selected brain areas [42]. Moreover, the common clinical practice of switching antipsychotic has been demonstrated to specifically perturb PSD molecules depending on the specific switch procedure (especially FGA to SGA, or vice versa) [37].

Therefore, antipsychotics may basically modify the architecture of the synapse through determining rearrangements of a complex calcium-regulated network with the final aim of integrating and routing both dopaminergic and glutamatergic signaling pathways to appropriate nuclear targets, therefore, finally impacting the intrinsic functions of synapses in selected brain areas [23]. Recent researches, indeed, pointed out the essential role of PSD molecules in the morphologic changes of dendritic spines induced by long-term antipsychotic treatment [65]. Moreover, a crucial role of microRNAs interactions with PSD molecules has been suggested in the radical synaptic plasticity rearrangements induced by antipsychotics [66].

### 2.2. Mood-Stabilizing Drugs

Several studies have demonstrated that mood stabilizing drugs, although very different amongst each other in chemical structure and mechanisms of action (especially lithium vs. valproate), may share a common impact on crucial components of post-receptor transductional pathways, such as the glycogen synthase kinase 3β (GSK3β) or the mitogen-activated protein kinases (MAPKs) [67]. Both lithium and valproate, by acting on those molecules, may control glutamatergic signaling via reducing membrane insertion of AMPA glutamate receptors through the regulation of GluR1 AMPA subunits phosphorylation status [68]. A similar action has been described for NMDA glutamate receptors, whose membrane insertion may be controlled by lithium through a direct action on NR2A phosphorylation and its interaction with PSD95 [69]. The impact of both lithium and valproate on GSK3β has been associated to a long-term increase in hippocampal synapse formation and connections, which could be directly correlated to mood stabilizing effects [70].

More recent studies demonstrated that chronic treatment with both lithium and valproate may affect the expression of genes coding for structural proteins of postsynaptic density, such as *Homer 1b/c*, *Shank* and *Inositol-1,4,5 trisphosphate receptors* (*IP3Rs*), which all represent a putative connection between glutamatergic and dopaminergic function as a further mood-stabilizing mechanism [71]. Moreover, when added to antipsychotics (such as it frequently occurs in clinical practice), mood stabilizers may induce a differential modulation of PSD molecules as compared to the effects of individual drugs in both cortical and subcortical brain regions [72]. Indeed, a growing body of evidence suggests a direct impact of mood stabilizers on dopaminergic neurotransmission [73], as well as that PSD molecules represents crucial dopamine-glutamate crossroads for the combined actions of mood stabilizers and antipsychotics [74].

Other mood stabilizers, such as carbamazepine and lamotrigine, although not sharing a common action on GSK3β as lithium and valproate, have been demonstrated to impact inositol pathways and MAPK cascades, which also represent core crossroads of dopamine-glutamate postsynaptic interplay [75,76].

### 2.3. Antidepressant Drugs

A complex dysfunction in synaptic plasticity of specific brain areas, such as the hippocampus and prefrontal cortex, is a well-known pathophysiological mechanism in depression [77]. Human studies have demonstrated that glutamate neurotransmission is impaired in cortical subregions in subjects suffering from major depression, with higher levels of NMDA receptors subunits in frontal cortex and even higher NMDA levels in parietal cortex in suicide completers, as well as reduced levels in dorsolateral prefrontal cortex and higher PSD-95 levels in anterior cingulate [78].

Chronic antidepressant treatment may induce morphological changes in the abovementioned brain areas through the direct modulation of PSD proteins. Indeed, fluoxetine has been demonstrated to increase the expression of hippocampal PSD95 and AMPA receptors subunit GluR1 via a mechanism involving TrkB receptors of BDNF [79]. Changes in both NMDA and AMPA receptors subunits provoked by chronic fluoxetine have been associated with forebrain up-regulation in dendritic spines and formation of mushroom-type spines [80]. Based on those observations regarding glutamate neurotransmission modulation by antidepressants, some studies have demonstrated that also serotonin receptors-modulating antipsychotics, such as lurasidone, may provoke hippocampal PSD proteins modulation and dendritic spines changes similar to that induced by fluoxetine [81]. Additionally, lurasidone has been demonstrated to exert antidepressant properties through a direct modulation of BDNF in prefrontal cortex of animal models of depressive states, thus suggesting a crucial impact of this drug on pathophysiologic neuroplastic mechanisms underlying depression [82,83]. Moreover, the concurrent administration of antipsychotics and SSRI antidepressants may induce synergistic modulation of specific PSD molecules, such as *Homer 1a*, thereby suggesting a fundamental crosstalk of serotonin and dopamine transductional pathways in the pathophysiology of depressive states [84]. Core molecules related to dopamine signal transduction, such as DARPP-32 have been reported to be significantly modulated by antidepressants, as well as the combination of antipsychotics and antidepressants may synergistically impact synaptic proteins related to energetic metabolism (for a review see: [74]). The essential role of dopamine modulation in depression, as well as the tight imbrication with pathophysiologic mechanisms also involved in schizophrenia, may be confirmed by the fact that dopaminergic antidepressant drugs, such as bupropion, have been demonstrated to be safe and effective in depressive states occurring in schizophrenic patients [85].

The above described glutamatergic postsynaptic mechanisms have been reported to be on the basis of the rapid antidepressant properties displayed by ketamine, an NMDA antagonist. Indeed, ketamine has been demonstrated to induce hippocampal PSD-95 modulation via the TrkB-BDNF pathway similar to those provoked by fluoxetine, but in a more rapid and unstable manner [86]. Moreover, the administration of lithium may potentiate the synaptogenic and antidepressant effects of ketamine by inhibiting GSK3β [87]. Recently, ketamine has been demonstrated to exert its antidepressant effects essentially via its metabolite hydroxynorketamine. Indeed, the R-enantiomer of hydroxynorketamine shows behavioral and biochemical antidepressants effects that seem independent from NMDA receptors’ inhibition, but involve a robust increase in AMPA receptors-mediated excitatory postsynaptic currents [88]. AMPA receptors, in fact, have been crucially correlated to the pathogenesis of both psychosis and mood disorders. Early deletion of AMPA GluR1 subunit may induce striatal hyperdopaminergia and behavioral abnormalities mimicking psychosis [89], as well as AMPA knockout mice exhibit increased learned helplessness, decreased serotonin and norepinephrine, and impaired glutamate neurotransmission, all features modeling depressive phenotypes [90]. However, only early impairment of AMPA function has been demonstrated to induce neuropsychiatric phenotypes, since post-adolescence-induced AMPA ablation results in normal behaviors in animal models [91,92]. These findings suggest that global dysfunctions of glutamatergic signaling that onset during early development are necessary to establish full depressive phenotypes. Indeed, ketamine has been demonstrated to have no effects on juvenile animals, because developmentally mature synapses are required to induce antidepressant responses [93].

Regarding the role of PSD proteins in the mechanisms of action of glutamatergic antidepressant drugs, since the modulation of both the GluN2A and GluN2B subunits of the NMDA receptor has been reported to individually exert antidepressant effects [94], a recent study demonstrated that the impairment of PSD-95 constitutive functions may impair NMDA-GluN2B-mediated antidepressant-like responses [95].

PSD molecules involvement in major neuropsychiatric disorders and their modulation by main psychopharmacologic drugs are summarized in Table 1.

## 3. Novel Putative Therapeutic Strategies Based on PSD Molecules Modulation

Based on the studies described above, it can be expected that, once the molecular mechanisms implicated in each distinct psychiatric disease would be at least partially unveiled, modulation of PSD molecules will be instrumental at restoring physiological synaptic functioning and, consequently, network connections at micro- and macro-circuit levels. However, as a challenge, the exact brain division, and even the cellular type, where these molecules operate to modulate definite behavioral conducts, and to putatively cause aberrations, has not been fully characterized. Nonetheless, an increasing body of evidence is locating PSD alterations in specific brain sites. In post-mortem brain samples from schizophrenia patients, significant changes in key PSD molecules (i.e., PSD-95, Homer 1a, Homer 1b, Preso) have been demonstrated in multiple brain regions, including the hippocampal CA1 region, the prefrontal cortex, and the olfactory bulb [96]. Exact delivery of PSD-targeting therapeutic agents in brain subdivisions or cell types implicated in disease pathophysiology may be crucial to maximize pharmacological efficacy and/or minimize untoward effects. Despite this field still being in its formative stage, recent novel technological approaches are attempting to overcome the challenges deriving from cell-type restricted drug delivery within the Central Nervous System (CNS), as in the case of the recent advent of nanomedicines, which provide potent tools to implement CNS targeted delivery of active compounds [97]. Great advances are mainly taking place in other biomedical fields, such as oncology or neurology. One such example is multiple sclerosis, where advanced drug delivery of the so-called disease-modifying therapies is under thorough investigation in order to decrease adverse effects, increase drug efficacy, and increase patient compliance through the direct targeting of pathologic cells [98]. Among advanced drug delivery systems, current studies are accounting for nanoparticles, microparticles, fusion antibodies, and liposomal formulations. Additionally, there is increasing efforts to delineate alternative routes of drug administration, such as the nasal route for systemic use, in order to cross the blood-brain banner (BBB) [99]. A recent report has demonstrated that the intranasal administration of a sertraline-conjugated small-interfering RNA (siRNA) was effective in silencing the expression and diminishing the biological function of the serotonin transporter (SERT) and evoked fast antidepressant-like responses in mice [100]. Overall these breakthrough drug delivery technologies are aimed at being applicable to multiple disease treatments in the CNS and may allow to precisely target pharmacological agents to PSD molecules in the cell types where their alterations participate in pathophysiology of psychiatric diseases.

As a third valuable challenge, it has to be mentioned that as PSD molecules share both architectural/functional and signaling roles in the post-synaptic neuron, they are simultaneously recruited in several downstream pathways, often representing sites of trans-activation and intersection among different intracellular cascades. Therefore, PSD molecules have pleiotropic biological roles and participate in several downstream pathways concurrently [101,102]. How to selectively target a distinct PSD protein-operated downstream pathway over the concurrent others is another relevant field of research. However, PSD molecule-targeting may more easily attain a modulation of selected downstream pathways compared to receptor-targeting pharmacological devices, possibly representing one of the major advantage of this novel strategy.

Given all the considerations above, modulation of PSD molecules as a therapeutic strategy in psychiatric diseases may occur either indirectly or directly. Indirect PSD molecule modulation intervenes as a consequence of the interaction of a pharmacological agent with their target non-PSD receptors. Indirect modulation may be regarded as an accessory molecular effect of a drug, which may, theoretically, be either incidental or desired. However, to date, there are no designed pharmacological agents whose primary or secondary desirable molecular effects are to specifically modulate PSD targets. On the other hand, multiple psychotropic pharmacological agents have been demonstrated to incidentally modulate gene and protein expression of PSD molecules [81], as also described in sections above. However, this approach has considerable shortcomings. Indeed, a clear demonstration that this effect is related to antipsychotic efficacy is still lacking. As a second cue, it has been only partially demonstrated that psychotropic agent-mediated modulation of expression of PSD molecules may translate in changes at the protein, functional, and behavioral levels. Despite these considerations, it has to be expected that PSD molecules participate in the biological consequences of psychotropic agent administration. This statement is supported by experimental evidence showing that similar modulations of target PSD molecules are the result of different therapeutic procedures. In a recent report, increased expression of the Homer1a in the medial PFC was associated with inhibition of depressive-like behaviors and was the ultimate molecular step of various antidepressant treatments, including sleep deprivation, up-regulation of adenosine 1 receptor, imipramine, or ketamine administration [103]. Evaluation of the pattern of expression of PSD molecules has also been suggested to provide information on augmentation or association therapeutic strategies. It has been reported that Arc expression is differentially modulated by haloperidol alone, or in association with minocycline, a synthetic second-generation tetracycline that has been proposed as an adjunctive treatment mostly for negative symptoms of schizophrenia [104]. In another study, it has been observed that the molecular changes in targeted PSD molecules are different in relation to the timing and the order in which antipsychotics are given [37]. Overall, these reports suggest that PSD molecules are key actors in the mechanisms of action of psychotropic agents and their modulation may represent one of the ultimate biological steps to trigger pharmacological effects of these drugs. In these terms, PSD molecule modulation may characterize other recently proposed therapeutic agents that act on glutamatergic transmission, such as: agonists at group 2/3 metabotropic glutamate receptors; positive allosteric modulators of type 5 metabotropic glutamate receptors; NMDA receptor modulators; AMPAkines; or glycine transporter inhibitors [2,105,106,107,108,109]. However, even in these cases, putative modulation of PSD molecules should be regarded as a secondary, accessory biological effect, whose actual involvement in these agents’ mechanisms of action is yet to be completely elucidated.

One intriguing novel therapeutic strategy should be represented by direct targeting of PSD molecules. In this case, the relevant challenge scientists should face is the intracerebral and intracellular localization of these molecules, which would constrain novel putative pharmacological agents to face a double barrier, i.e., the blood-brain barrier and the cell membrane. In fact, current agents in neurology and psychiatry are able to cross the blood-brain barrier, due to their high lipophilicity. Only recently technological advance has made it possible to develop pharmacological devices with the aptitude to trespass the neuron cell membrane and interact with protein or genic elements [110]. NA-1 is a recently described PSD-95 small-peptide inhibitor, which prevents PSD-95 interaction with the NR2B subunit of NMDA receptors, thereby uncoupling NMDA receptors from nNOS-mediated downstream neurotoxic signaling pathways [111]. Notably, this uncoupling does not affect NMDA receptor-mediated excitatory neurotransmission in the brain [111]. NA-1 has been previously tested in rat and non-human primate models of transient focal ischemia and stroke [112,113]. Subsequently, this agent has been studied in clinical stroke treatment in humans [114] and is currently undergoing phase III clinical trials.

NA-1 represents a paradigmatic example of a pharmacological agent, which directly modulates PSD molecules for the treatment of neuropsychiatric disease. The possibility to expand the therapeutic use of NA-1 to behavioral diseases appears minimal, since it targets a very restricted downstream pathway. However, this example opens the way to the possibility of designing small molecule inhibitors to interact with other portion of PSD-95, or with other PSD molecules, in order to disrupt the protein-protein interactions and modulate selected downstream pathways implicated in behavioral disease mechanisms.

## 4. Conclusions

PSD molecules represent crucial crossroads for multiple receptor signaling involved in the mechanisms of action of the most frequently used psychopharmacologic drugs. Their close intermingling receives, elaborates, and converges multiple signals to the appropriate nuclear targets, in order to finely modulate synaptic rearrangements in response to neural activity. However, PSD molecular mechanisms are so sophisticated and complex that their knowledge is only partial at the moment. Further studies will putatively permit the development of “synapse-targeting” psychopharmacotherapeutics, which could finally bypass the pharmacodynamics and pharmacokinetic problems that currently jeopardize a fully-effective psychopharmacologic treatment.

## Figures and Tables

**Table 1:** PSD molecules involvement in major neuropsychiatric disorders and their modulation by main psychopharmacologic treatments.

PSD Molecule	Involvement in Major Neuropsychiatric Disorders	Modulation by Psychopharmacologic Drugs
Homer 1	-Schizophrenia [2,65,66,115,116,117,118] -Bipolar Disorder [9] -Major Depressive Disorder [119,120] -Drug addiction [121,122,123,124,125,126,127,128] -Chronic inflammatory pain [129,130] -Fragile X Syndrome [131] -Alzheimer’s Disease [132] -Parkinson’s Disease [133,134] -Traumatic brain injury [135,136]	-*Homer 1a* may be differentially modulated by both first generation and second generation antipsychotics tightly depending on their own individual receptor profile [42,61,62,137,138] -The mood stabilizers lithium and valproate have scarce effects on *Homer 1a* expression, whereas they deeply impact synaptic structure conformation by modulating constitutive *Homer 1b/c* gene expression [71] -Combination of antipsychotics and mood stabilizers elicits changes in *Homer 1a* gene expression that are substantially different from those induced by these drugs individually administered [72] -Antidepressants and serotonin-modulating antipsychotics induce peculiar cortical expression of *Homer 1a* in brain regions relevant for negative and cognitive symptoms of schizophrenia [62,84]
Homer 2	-Schizophrenia [139] -Alcohol abuse [140,141,142]	-Chronic haloperidol and clozapine administration may induce overexpression of *Homer 2* in lateral septum in animal models [62]
Homer 3	-Cerebellar ataxias [143,144]	
PSD-95	-Schizophrenia [46,145] -Autism Spectrum Disorders [46,146] -Bipolar Disorder [9,147] -Major Depressive Disorder [148]	-Lurasidone and fluoxetine decrease PSD-95 expression in prefrontal cortex and hippocampus [81] -Olanzapine and aripiprazole may reverse the immobilization stress-induced decrease in PSD-95 levels in frontal cortex [149] -PSD-95 is crucial for serotonin 5HT2A and 5HT2C receptors expression and abolishing its expression in knockout animals impairs atypical antipsychotics effects [64] -Ketamine impacts PSD-95 expression in cortical and striatal regions [150], and PSD-95 seems to be crucial for ketamine antidepressant effects [95]
Shank	-Schizophrenia [44,151,152] -Autism Spectrum Disorders [153,154,155]	-The mood stabilizers lithium and valproate may down-regulate *Shank* cortical expression when chronically administered in animal models [71]
GSK3β	-Schizophrenia [156,157,158,159] -Major Depressive Disorder [158,159] -Bipolar Disorder [160,161]	-Aripiprazole activates GSK3β signaling in prefrontal cortex and nucleus accumbens, whereas haloperidol activates GSK3β signaling only in nucleus accumbens [162] -Paliperidone exerts protective effects on neurons via decreasing glutamate-induced overactivation of GSK3β signaling [163] -Clozapine may increase GSK3β signaling in prefrontal cortex, but not in striatum, where it is activated by haloperidol [164] -Fluoxetine and imipramine have scarce effects on GSK3β signaling [164] -The inhibition of GSK3β signaling seems to be a crucial mechanism explaining mood stabilizing effects of lithium [165] -Valproate inhibits metamphetamine-induced hyperlocomotion via decreasing GSK3β activity [166]
DISC1	-Schizophrenia [157,167] -Bipolar Disorder [168,169]	-Atypical antipsychotics may increase cortical expression of *DISC1*, whereas typical antipsychotics have no effects [170] -Specific genomic variants in *DISC1* gene in humans have been associated to ultra-resistance to antipsychotic treatment [171]
CAMKII	-Schizophrenia [172] -Major Depressive Disorder [173]	-Clozapine-induced increase in prefrontal cortex activity is crucially mediated by CAMKII-NMDA receptor interactions [174] -Clozapine, haloperidol and risperidone may decrease *CAMKII* expression in striatum in animal models [175] -CAMKII is essential for clozapine-mediated effects on conditioned avoidance responses in animal models [176] -Fluoxetine may induce changes in *CAMKII* promoter [177]
